# Petrographic and geochemical study of Precambrian and Paleozoic rocks in the western Anti-Atlas belt, Morocco: Facies, weathering, provenance and economic exploitation

**DOI:** 10.1016/j.heliyon.2024.e33290

**Published:** 2024-06-19

**Authors:** Mohamed Mahmoud Sebbab, Mehdi Ousbih, Mohamed En-Nasiry, Abdelhadi El Ouahidi, Kamal Abdelrahman, Abdessamad El Atillah, Md Galal Uddin, Armel Zacharie Ekoa Bessa, Mohammed S. Fnais, Agnieszka I. Olbert, Mohamed Abioui

**Affiliations:** a Spaces, Societies, Environment, Planning and Development Laboratory, Department of Geography and Planning, Faculty of Languages, Arts and Human Sciences- Ait Melloul, Ibnou Zohr University, Ait Melloul, Morocco; b Department of Earth Sciences, Faculty of Sciences, Ibnou Zohr University, Agadir, Morocco; c Department of Geology & Geophysics, College of Science, King Saud University, Riyadh, Saudi Arabia; d School of Engineering, University of Galway, Galway, Ireland; e Ryan Institute, University of Galway, Galway, Ireland; f MaREI Research Centre, University of Galway, Galway, Ireland; g Eco-HydroInformatics Research Group (EHIRG), Civil Engineering, University of Galway, Galway, Ireland; h Department of Earth Sciences and Environment, Higher Teacher Training College, University of Bertoua, Bertoua, Cameroon; i MARE-Marine and Environmental Sciences Centre - Sedimentary Geology Group, Department of Earth Sciences, Faculty of Sciences and Technology, University of Coimbra, Coimbra, Portugal; j Laboratory for Sustainable Innovation and Applied Research, Universiapolis—International University of Agadir, Agadir, Morocco

**Keywords:** Petrography, Geochemistry, Western Anti-Atlas, Facies analysis, Weathering, Provenance

## Abstract

Detrital and volcanic-detrital rocks from the Ifni Buttonhole and Lakhssas Plateau were analyzed to determine their provenance, compositional maturity, and alteration source. Geochemically, the sediments were classified as arkoses, lithic arenites, grauwackes, sandstones, lithic arenites, and Fe-rich sands, indicating low compositional and mineralogical maturity. A high average SiO_2_ concentration and low Al_2_O_3_ were consistent with a low abundance of shale and clay components. The geochemical signatures of the detrital and volcano-detrital (RDVD) rocks indicate that they have undergone a moderate to low degree of chemical alteration. The CIA study also suggests that the granitic, granodioritic rocks represent the source provenance which, during weathering and transport, supplied the detritus to the supra-crustal units. The major trace element data suggest that these rocks are largely derived from felsic igneous rocks, namely granitoids, with a minor contribution from intermediate sources. The carbonate rocks do not represent a wide variety of facies: dolomitic limestone, calcareous limestone, and calcaro-dolomitic chert. Calcitic and dolomitic samples show a linear increase in SiO_2_, regardless of their CaO/LOI ratio values, which remain relatively constant. The highest SiO_2_ contents are observed in the calc-dolomitic chert. Geochemical analysis of RDVD from the Ifni buttonhole determined their origin, maturity, and alteration. Major oxides decreased with higher silica content, indicating quartz control. Samples, formed under semi-arid conditions, show maturity under stable deposition. They suggest a felsic, recycled source, with moderate alteration and zircon enrichment during recycling. In the study area, limestones and dolomites serve as materials applicable in the building sector, suitable for all types of concrete. The Taliwine Formation harbors Lower Cambrian dolomites and limestones, ideal for mosaic aggregates. Described as variable in color, compact, homogeneous, very hard, and resistant to alteration, the plutonic rocks form prominent peaks. They exhibit both subalkaline characteristics in granitoids and an alkaline trend in dolerite dykes. Most samples display minimal alteration, indicating the reliability of their major element compositions for geochemical analyses. These granitoids constitute valuable deposits for ornamental and building rock purposes.

## 1 Introduction

Economic growth in recent years has led to a great deal of interest in the use of natural stone in construction work. Exploration activities have increased and new quarries of dimension stone have been opened. Granitoids and marbles, for example, are widely used because of their aesthetic quality and hardness. Ornamental rocks are also increasingly used in construction. The exploitation of granites and marbles is a very important sector for socio-economic development [1]. However, the methods used both for the extraction of blocks in the quarries and the cutting and finishing techniques used in the manufacturing workshops should follow the evolution of modern techniques. Thus, investors in this sector will have to equip themselves with modern extraction equipment and recruit qualified and specialized personnel; this will certainly encourage national consumption of marble and boost exports. It would therefore be interesting to develop the resource potential of the Sidi Ifni region in this field to meet the growing demand.

The relationship between the compositional attributes of siliciclastic sediments and their tectonic setting and provenance has been extensively investigated by numerous workers e.g., Refs.[2,3]. Both petrographic and geochemical analyses of siliciclastic rocks have been pivotal in elucidating aspects such as provenance, weathering, climate, and tectonic setting of source areas e.g., Refs.[4,5]. Petrographic and mineralogical examinations of detrital grains have been instrumental in the systematic classification of sandstones and in discerning their provenance and tectonic setting e.g., Refs.[2,[6], [7], [8], [9], [10], [11]]. Additionally, the geochemical analysis of siliciclastic rocks has provided valuable insights into the interpretation of tectonic settings and depositional environments of clastic rocks e.g., Refs.[3,9,[12], [13], [14], [15], [16], [17]].

The rocks exposed in the Ifni inlier and Lakhssas Plateau of the Anti-Atlas region exhibit Paleoproterozoic to Paleozoic age characteristics and are predominantly comprised of clastic materials interbedded with carbonates e.g., Refs.[[18], [19], [20], [21]]. Lower Paleozoic formations, primarily consisting of sandstones, overlay Precambrian basement rocks unconformably, with varying thicknesses owing to the irregular surfaces over which deposition occurred [22].

Although the quarry operator necessarily has a fairly good geological knowledge of his site, the primary purpose of this knowledge is to produce products that meet the needs of the market. Consequently, this knowledge is generally limited to the physical characteristics of the extracted rocks (accessibility, volume, hardness, structure). The yield of a quarry in granite, for example, depends on the petrographic nature of the rock, its degree of weathering and cracking, and the frequency of blocks to be extracted, in addition to the cost of extraction and transport of the material.

Numerous high-quality stone deposits are available in the Ifni Buttonhole and Lakhssas Plateau sites. These natural stones can be used in the form of ashlars, rubble, cladding of facades, floors, and walls, landscaping of patios and pavements, paving of alleys, and manufacture of carved pieces. The natural stones used in the western part of the Anti-Atlas and the High Atlas are classified according to their lithological nature and age; we distinguish between igneous rocks (Precambrian granitoids of Sidi Ifni and Kerdous), sedimentary rocks (Triassic sandstones of Argana and Cretaceous limestones of the hills of the southern Atlas zone) and metamorphic rocks (Paleozoic slate schists and quartzo-schists and Cambrian marbles of Lakhssas and Imi m'Qorn). The wide variety of material choices is a distinctive advantage.

This study aims to analyze the geological features of the Ifni Buttonhole and the Lakhssas Plateau. We investigate their Precambrian basement overlaid with Neoproterozoic to Cambrian deposits, followed by Cretaceous and Quaternary layers. Our focus lies in understanding the petrographic and geochemical properties of various facies within these formations to unravel their provenance and tectonic setting. Through a petrographic and geochemical study, we aim to uncover facies patterns, weathering processes, provenance significance, and potential economic opportunities.

## 2 Geological setting

### 2.1 Regional geology: Anti-Atlas

**Fig. 1:**
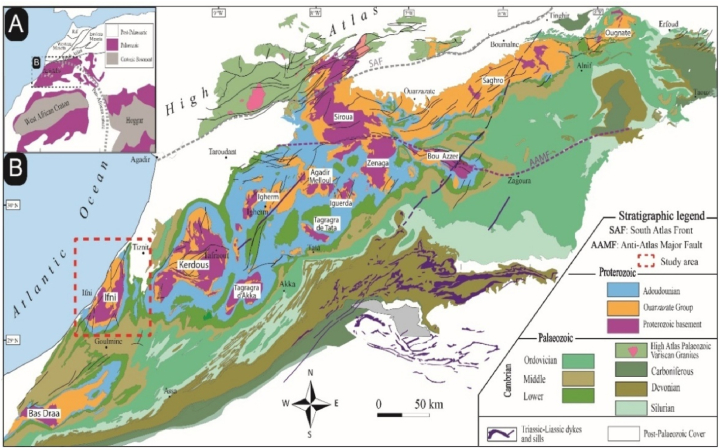
Schematic geological map of the Precambrian buttonholes of the Anti-Atlas chain (modified from Gasquet et al. [44]).

Located at the northern edge of the West African Craton (WAC), the Anti-Atlas is bounded to the north by the High Atlas Mountains e.g., Refs.[[23], [24], [25]] (Fig. 1a). This structural domain, located south of the High Atlas, is about 750 km long and 250 km wide, running SW-NE from the Atlantic Ocean in the west to Algeria in the east (Fig. 1b). It consists of Precambrian, crystalline, crystalline and volcano-detrital formations which outcrop in buttonholes in an essentially carbonate cover, the upper part of which, at least, is Cambrian [[26], [27], [28], [29]]. The numerous studies on the Precambrian of the Anti-Atlas and more particularly those of Choubert [30], Choubert and Faure-Muret [31], Leblanc [32], and Leblanc and Lancelot [33] have allowed this region to be subdivided into three structural domains: (i) the central Anti-Atlas on either side of and along the major accident of the Anti-Atlas or Leblanc's suture zone, (ii) the western Anti-Atlas to the south-west of this accident, and (iii) the eastern Anti-Atlas to the north-east of this accident. For this author, the southwestern ancient domain corresponds to the western Anti-Atlas. It represents the frontal edge of the West African Craton (WAC). The Anti-Atlas consists of terrains of Lower Proterozoic age, cratonised during the Eburnian orogeny and unconformably overlain by Upper Proterozoic calcaro-quartz sediments, themselves unconformably overlain by Terminal Proterozoic volcanic terrains.

The Anti-Atlas was the location of the Eburnian, Pan-African, and Hercynian orogenies [34,35], which succeeded each other in time during the Paleoproterozoic, Mesoproterozoic, Neoproterozoic, and Paleozoic respectively [36]. According to the lithostratigraphic classification of Thomas et al. [37], the Proterozoic basement consists of the oldest rocks of the Anti-Atlas. It corresponds to the major complex formerly called "Precambrian I: PI" by Choubert [38]. It is composed of various magmatic and metamorphic lithologies of different degrees (meta-sediments, schists, para-gneisses, granites, orthogneisses, and migmatites). The Neoproterozoic is represented everywhere in the Anti-Atlas and corresponds to various formations: limestones, quartzites, schists, basic and ultrabasic rocks, volcanic and plutonic rocks, spilites and keratophyres, tuffs, flyschs, molasse, ignimbrites, and andesites, granitoids [33,34,37,[39], [40], [41], [42], [43], [44], [45], [46]]. The Paleozoic terrains form a platform cover over the ancient terrains, and their structuring during the Hercynian orogeny will give the current shape of the Anti-Atlas. From a geomorphological point of view, erosion has sculpted a buttonhole relief in the Proterozoic terrains under a Paleozoic cover.

### 2.2 Local geology: Ifni buttonhole and Lakhssas Plateau

The Moroccan Anti-Atlas is made up of numerous ante-Cambrian buttonholes where Proterozoic granitoids with terminal Neoproterozoic and Paleozoic coverings are outcropping. The Ifni buttonhole forms the western end of the Anti-Atlas, which in turn forms the northern edge of the West African Craton [31].

**Fig. 2:**
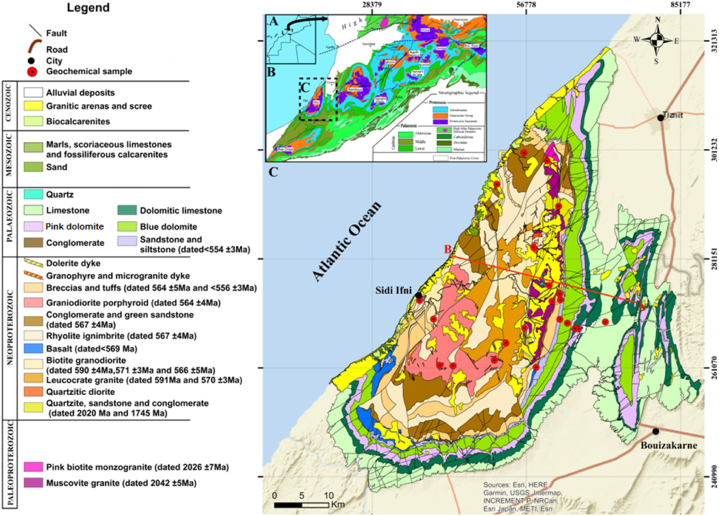
Simplified geological map of the Ifni buttonhole and Lakhssas Plateau (modified after Benziane et al. [75]).

**Fig. 3:**
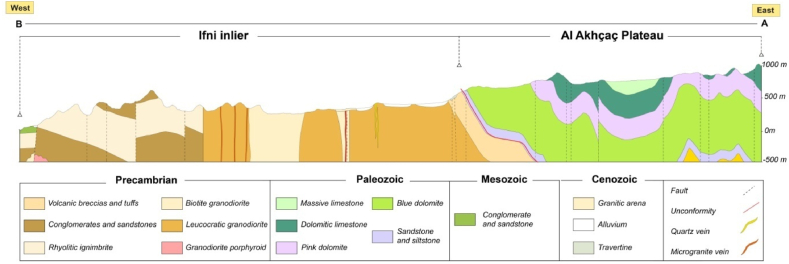
Geological section E-W (the red cross-section A-B in Fig. 2), crossing the Ifni buttonhole and the Lakhssas Plateau. (For interpretation of the references to color in this figure legend, the reader is referred to the Web version of this article.)

The geological history of the Ifni Buttonhole begins in the Paleoproterozoic and continues through the Middle Neoproterozoic, Late Neoproterozoic, Paleozoic, Mesozoic, and Cenozoic [18,[23], [24], [25],^37^,[47], [48], [49], [50], [51], [52], [53]]. The geodynamic evolution of the Ifni Buttonhole is probably subdivided into six stages (Fig. 2a, b, c): (a) Orosirian (Paleoproterozoic), (b) Cryogenian (Middle Neoproterozoic), (c) Ediacaran (Upper Neoproterozoic), (d) Lower Paleozoic, (e) Hercynian outcrops as quartz veins, but also present by main tectonics associated with metamorphism, and (f) Lower Cretaceous and Cenozoic formations. The lfni buttonhole is formed by various granitoids surrounded by volcanic and volcano-sedimentary formations of the Neoproterozoic age. According to available geochronological data [18,[54], [55], [56]], the Paleoproterozoic has been dated to the Orosirian, an event related to the Eburnian orogeny, and includes the Alouzad suite granites, outcropping over a small area in the east of the massif [22]; the Lower Ediacaran is composed of a succession of volcanics, volcanoclastites, breccias, and conglomerates deposited on the basement and intrusive granitoids; the Upper Neoproterozoic is composed of acidic and then basic volcanics and dykes that overlie or cut the underlying ensemble. The geochronological and geochemical results are essential for a regional comparison with the other Anti-Atlas buttonholes [18,57]. Moving eastwards in the buttonhole, overlying these units are the latest Neoproterozoic, Cambrian (Taroudant and Tata Groups), and Ordovician (Fig. 3).

The Ifni Precambrian buttonhole belongs to the western end of the Anti-Atlas, a vast bulge that abuts the High Atlas by the powerful volcanic mass of Jbel Siroua. This buttonhole is made up of sedimentary and volcano-sedimentary formations that are not very or not tectonized, which are embedded in the north, east, and south under the carbonate cover. The main lithostratigraphic formations individualized in this buttonhole are crossed by successive occurrences of plutonic rocks. The major part of the studied region is constituted of plutonic rocks of granitic composition. The Lakhssas Plateau is bordered by two buttonholes, Ifni to the west and Kerdous to the east. The plateau corresponds to a large synclinorium of Cambrian carbonate rocks showing an anticlinal structure in its core. In the central part of the plateau, gravity and magnetic data suggest the presence of an uplifted basement block beneath the deformed limestone massif [21,58]. This structure is interpreted as a horst produced by Late Proterozoic extension and then reversed during Variscan compression [34,58].

## 3 Analytical methods and scientific approach

A sampling campaign in the Lakhssas Plateau and the Ifni buttonhole was carried out during June and July 2021. During this campaign, we proceeded to a description of different outcrops and rock sampling. Concerning the rock samples, a total of 24 samples from different geological outcrops were collected. These rocks were subjected to a detailed petrographic study and an elementary geochemical analysis. Twenty-five thin sections were made from these samples.

The samples were shipped by the Faculty of Languages, Arts and Human Sciences (Ibnou Zohr University, Morocco). The thin sections were made in the MANAGEM center (Morocco). The petrographic study of the thin sections was carried out in the Department of Earth Sciences (Faculty of Sciences, Ibnou Zohr University, Morocco). The geochemical study using the wavelength dispersive X-ray fluorescence spectrometry (WD-XRF) technique was carried out at the AREMINEX laboratory of the MANAGEM center (Morocco). The map in Fig. 2a, b, c shows the distribution of the sampling points in the study area.

Loss on Ignition (LOI), measured on these powders, gives us information on the state of alteration of the rocks and consists of calcining about 1g of sample contained in a porcelain crucible in an oven at about 1050 °C for 1 h. The mass before and after calcination is measured, and calcination can then cause a loss of mass of the sample due to the loss of volatiles, and/or a gain in mass if there is the sufficient presence of Fe^2+^ which oxidizes to Fe^3+^ [59]. This protocol allows the degree of weathering and the amount of water and carbonates present in the sample to be quantified. The higher LOI content, the more hydrated secondary minerals appear.

All igneous rocks have some amount of volatile elements. These elements are usually expressed in what is called a Loss on Ignition (LOI %) analysis expressed in percentage. It is calculated as the loss of mass of a sample after heating to about 1000 °C [60]. The value obtained represents the sum of the volatile elements present in the rock. The sum of the major oxides can therefore be calculated on an anhydrous basis (or rather without volatiles since the loss on ignition includes elements other than water). This calculation is carried out in the following way:

Calculation « anhydrous »: element * (100 + LOI) /100

To better appreciate the impact of secondary processes, we used the "Oxides LOI" diagrams. This dry recalculation is necessary when the rocks are altered and therefore the analyses no longer reflect the original chemical compositions of the rocks.

## 4 Results

### 4.1 Description of outcrops

#### 4.1.1 Plutonic rocks

**Fig. 4:**
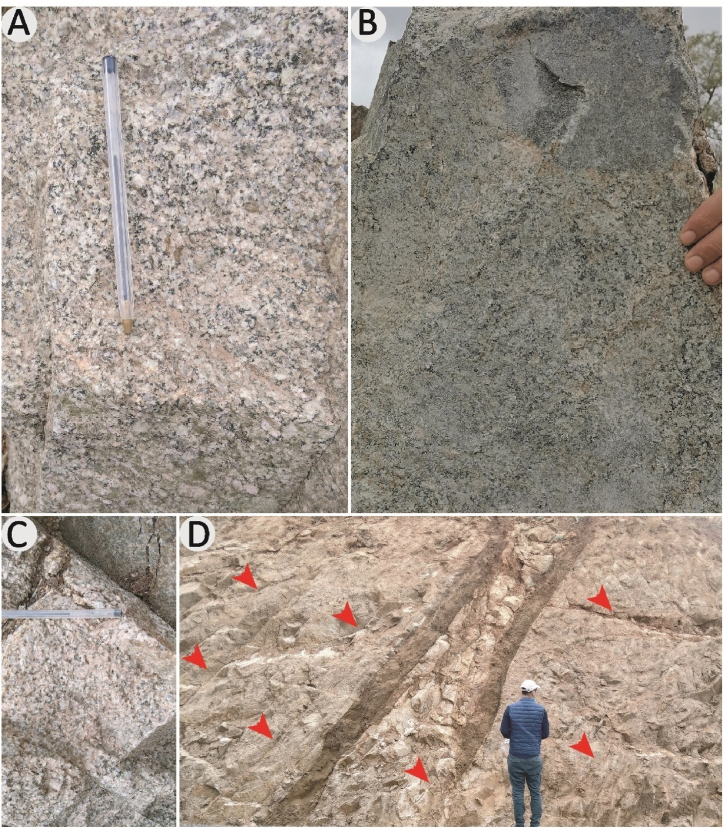
a & c, granite contains an enclave of magmatic rock; **b**, plutonic rock shows a greenschist facies metamorphic paragenesis; **d**, granite crossed by a network of fractures.

The Ifni inlier exhibits numerous outcrops of granitoid massifs, with a prevalence of granitic lithologies. The Neoproterozoic plutonic includes the Taoulecht granite, Tirhit granodiorite, Mirleft granodiorite, Tioughza granite, Ifni granodiorite and Mesti granodiorite. Paleoproterozoic intrusions include the Alouzad granite and the Sahel biotite monzogranite. At outcrop, the plutonic rocks observed exhibit chlorite, biotite, quartz, albite, and epidote greenschist facies metamorphic paragenesis (Fig. 4a). They harbor abundant rounded enclaves (Fig. 4b), partially assimilated from magmatic, sedimentary (sandstone and siltstone), or volcanic (rhyolitic tuff) origins. The plutonic rocks described are of different colors, pink and grey, compact, homogeneous, and very hard, difficult to alter, and forming high peaks in the relief. Sometimes they contain subparallel fractures (Fig. 4c) and dykes (Fig. 4d) which are often the feeder pipes of different volcanic rocks.

#### 4.1.2 Detrital and volcano-detrital rocks

**Fig. 5:**
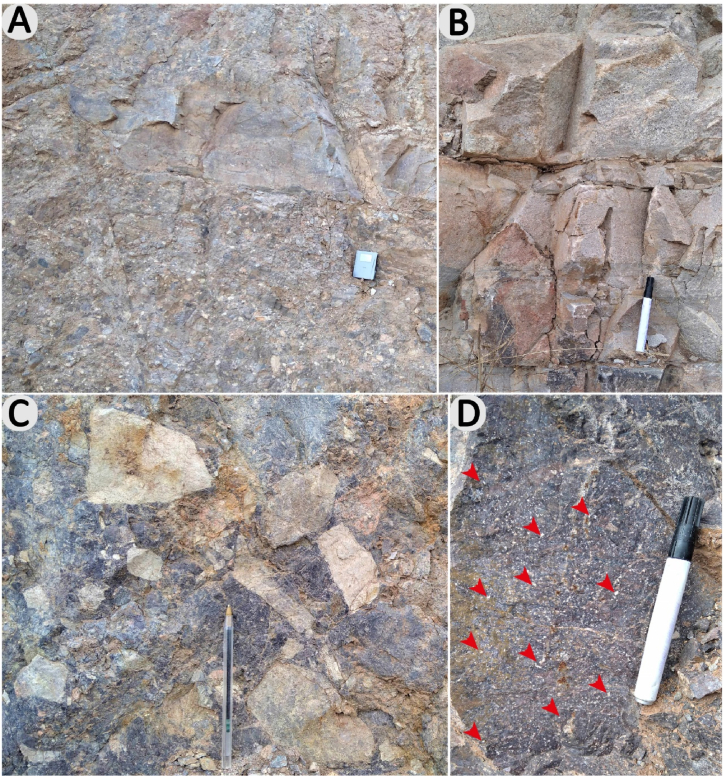
a, Intercalation of breccias and tuffs in thick benches; **b**, Alternations between lithic tuffs with lapillis and coarse tuffs in well-stratified decimetric troughs; **c**, Volcanic breccia with rock fragments, generally angular in shape; **d**, Massive crystalline tuffs, beige in color, containing quartz and feldspar minerals. (For interpretation of the references to color in this figure legend, the reader is referred to the Web version of this article.)

The detrital and volcano-detrital rocks e.g., Refs.[34,61] are weakly metamorphic, comprising a detrital series with intercalated ignimbrites of volcanic breccias, conglomerates, sandstones, and tuffs. The tuffs are well stratified with an alternation between lithic tuffs, lapillis, and crystalline tuffs with lithic clasts (Fig. 5a). The breccias and tuffs are interbedded as thick banks, outcropping in bands several hundred meters wide (Fig. 5b). In these coarse tuffs, the proportion of clasts is very variable. The lithic tuffs are made up of angular, centimeter-sized rock fragments, but also of numerous rounded blocks set in a volcanic matrix and generally constitute 40–60 % of the rock. The breccias contain mainly volcanic clasts, angular in shape, rarely rounded and ranging in size from a few millimeters to several decimeters (Fig. 5c). The conglomerates and epiclastic sandstones are interspersed with rhyolitic tuffs (Fig. 5d) and kinerites. The conglomerates are polygenic and heterometrically composed of quartzites, sandstones, and rhyolites, which vary in size from centimeters to decimeters. The matrix is essentially sandstone-tuffaceous and the cement is siliceous.

#### 4.1.3 Carbonate rocks

**Fig. 6:**
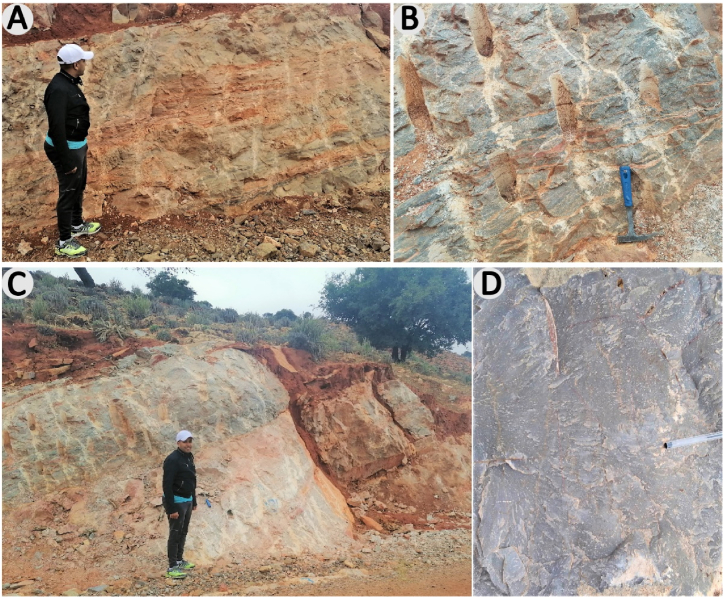
a, Stacked carbonate layers and calcareous-marl interbanks; **b**, Limestone layer showing stromatolitic laminations and dissolution cavities; **c**, Massive limestone banks, metric strength; **d**, Massive grey limestone.

Within the Lakhssas Plateau, the lithological composition is primarily characterized by Cambrian carbonate, calcareous, and dolomitic formations, heavily influenced by karstic morphogenesis processes. The most dominant rocks are the archaeocyathid limestones, the grey-blue limestones, and the lacustrine limestones. The lithological succession is generally monotonous and corresponds to a decametric to multi-decametric stacking of carbonate and inter-bank calcareous or pelitic beds (Fig. 6a). The dolomitic layers are blue-grey or pink in color or white with a grey patina. They show stromatolitic laminations, as well as dissolution cavities (Fig. 6b). The rock is massive, showing large benches (Fig. 6c). The joints between the beds are generally dry and sometimes consist of thin dolomitic or marly beds. The limestones are blue-black, grey, or white and contain archaeocyathids (Fig. 6d). The interbed joints generally appear empty and sometimes consist of thin dolomitic or marly beds.

### 4.2 Petrography

**Fig. 7:**
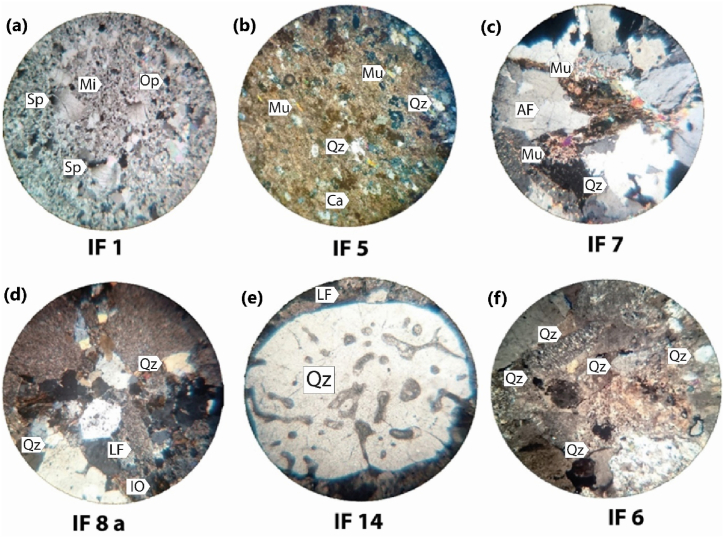
Photomicrographs of thin sections illustrating typical microstructures of various rock types, encompassing carbonate. **a**, IF1: Limestone – Sparite (Sp), micrite (Mi), muscovite (Mu), opaque (Op); **b**, IF5: Quartz limestone – Calcite (Ca), quartz (Qz), muscovite, opaque minerals (Op), Magmatic; **c**, IF7: Granite - Quartz, alkali feldspar (AF), plagioclase feldspar, muscovite, sericite, chlorite, biotite (Bt); **d**, IF8a: Ignimbrite - Quartz phenocrysts, rhyolitic rock fragment, chlorite, iron oxides, Detrital, and Volcano-Detrital; **e**, IF14: Conglomerate - Quartz, lithic fragments (LF), opaque minerals; **f**, IF6a: Conglomerate - Plagioclase, olivine, opaque minerals, muscovite, iddingsite rocks.

From a petrographic point of view (Fig. 7a–f), the macroscopic study consists of determining the type of facies to be exploited and its degree of homogeneity, the structures it presents, and the absence or presence of enclaves and/or injection veins. Table 1 displays cases of the various magmatic and sedimentary facies analyzed within the Ifni buttonhole and the Lakhssas Plateau zones. These have undergone geochemical analysis. Fig. 7 presents microscopic views of the distinct facies, including limestone (Fig. 7a), quartz limestone (Fig. 7b), granite (Fig. 7c), Ignimbrite (Fig. 7d), conglomerate (Fig. 7e), and dolerite (Fig. 7f). Additional macroscopic descriptions are provided in Table 1. Subsequent paragraphs will explore the detailed analysis of each facies type.

**Table 1:** Summary of the mineralogical composition and texture of different studied rocks.

Samples	Facies	Mineralogy	Texture and/or structure
IF 1	Limestone	Sparite (Sp), micrite (Mi), muscovite (Mu), opaque (Op)	Micritic
IF 2	Limestone	Sparite, microsparite, micrite, quartz (Qz), orthoclase (AF)	Mesosparitic
IF 3	Limestone	Sparite, microsparite, quartz, orthoclase, opaque minerals	Mesosparitic
IF 4	Pink dolomite	Dolomite, calcite, quartz, oxides, opaque minerals (Op)	Equigranular sparitic
IF 5	Quartz limestone	Calcite, quartz, muscovite, opaque minerals	Bedded
IF 6a	Conglomerate	Quartz, lithic fragments, opaque minerals	Clastic
IF 6c	Lapilli tuff	Quartz, alkali feldspar, sericite, oxides and opaque minerals	Grenue
IF 7	Granite	Quartz, alkali feldspar, plagioclase feldspar, muscovite, sericite, chlorite, biotite	Porphyry
IF 8	Lapilli tuff	Quartz, plagioclase feldspar, alkali feldspar, lithic fragments, opaque minerals	–
IF 8a	Ignimbrite	Quartz phenocrysts, rhyolitic rock fragment, chlorite, iron oxides	Porphyry microlitic
IF 8b	Dolerite	Plagioclase, opaques, chlorite, secondary quartz	Doleritic
IF 9	Granite	Quartz, alkali feldspar, biotite, muscovite, chlorite	Porphyry
IF 10	Granodiorite	Quartz, orthoclase, biotite	Porphyry
IF 11a	Crystal tuff	Quartz, calcite, rock fragment	–
IF 11b	Ignimbrite tuff	Phenocrysts of alkali feldspar and plagioclase, fragment of rhyolitic rock	–
IF 12	Rhyolite	Quartz, orthoclase and plagioclase, opaque minerals, spherulite	Porphyry microlitic
IF 13	Granodiorite	Quartz, altered orthoclase, biotite	Grenue
IF 14	Conglomerate	Quartz, lithic fragments (LF), opaque minerals	Clastic
IF 15	Monzogranite	Quartz, orthoclase, plagioclase feldspar, biotite, muscovite, sericite, opaque minerals	Grenue
IF 16	Dolerite	Plagioclase, olivine, opaque minerals, muscovite, iddingsite	Doleritic
IF 17	Quartzite	Quartz	Grenue
IF 18	Granite	Quartz, orthoclase, plagioclase, biotite, sericite	Porphyry
IF 19	Carbonated cement sandstone	Quartz, calcite, oxides, micrite	Porphyry

The conglomerates (Fig. 7e) are composed of rhyolites, sandstones, and quartzites. The matrix is sandstone-tuffaceous and the cement is siliceous. The size of the elements varied from centimeters to decimeters. The elements are rounded or angular and their classification is poor. The sandstones have a felsitic matrix containing xenocrysts of mottled plagioclase, rarely alkali feldspar, and angular quartz. Most feldspar xenocrysts are sericitized. The rare muscovite and opaques form interstitial grains. Tuffs lapilli form thin levels interspersed in breccias and lapilli tuffs which show a sericite-rich, recrystallized tuff forming two beds. The recrystallized tuff consists of a sericite and felsite matrix with corroded or recrystallized volcanic quartz xenocrysts and biotite, opaque or sericite xenoliths (probably alkali feldspar pseudomorphs) and felsite matrix and opaque rich xenoliths that reflect an ignimbritic structure e.g., Refs.[34,61]. The fine bedding shows a fine, homogeneous sericite matrix packing small quartz, biotite, and opaque elements. The ignimbritic tuffs (Fig. 7d) show fluidity figures or lava flow, the rock matrix is fine, felsitic, and contains xenocrysts of mottled plagioclase and sanidine. The matrix shows remnants of recrystallized pearlites which are surrounded by opaques and sericite. Some feldspars and quartz aggregates are bordered by opaques which emphasize the fluidity of the rock.

The granitoids (Fig. 7c) have a porphyroid aspect with large, pink, or white feldspars, several mm in size and rich in muscovite. They contain numerous elongated enclaves. Porphyroid granites contain large quartz and feldspar crystals that are cracked, broken, and oriented. It is muscovite and biotite porphyroid granite with two generations of mineral crystallization. The dolerite dyke under the microscope is a horny mesostasis of epidote, green biotite, quartz, albite, plagioclase, sericite, and sub-automorphic sphene. The felsitic mesostasis of automorphic plagioclase in trachytic structure contrasts with a chlorite recrystallized matrix. Plagioclases clearly show their subvolcanic origin.

Carbonate rocks containing dolomite crystals are large. Small calcite crystals of any shape surround the dolomite crystals. There are quartz grains with a size not exceeding 200 μm. Clear sub-automorphic quartz grains of about 1 mm are corroded by calcite. In the dolomitic samples, there are dolomitic interlocking sparitic crystals, micritic pellets in clusters, or well-isolated and angular to rounded quartz grains corroded by calcite. Some quartz has blackish inclusions of oxides. Hematite in granules and patches sometimes colors the rock red. Sparitic calcite corrodes the quartz and fills the space between the pellets. Calcite attacks the dolomite and crystallizes between the dolomite crystals. The limestones show sparitic calcite crystals ranging in size from 100 to 500 μm (Fig. 7a). The calcite crystals are interlocking. Small calcite crystals of any shape surround the large crystals. There are rounded quartz grains with a size of no more than 200 μm, which are in clusters or isolated. The quartz grains are corroded by calcite. The bottom of the rock consists of microsparite to sparitic calcite (Fig. 7b).

### 4.3 Geochemistry

#### 4.3.1 Magmatic rocks

**Fig. 8:**
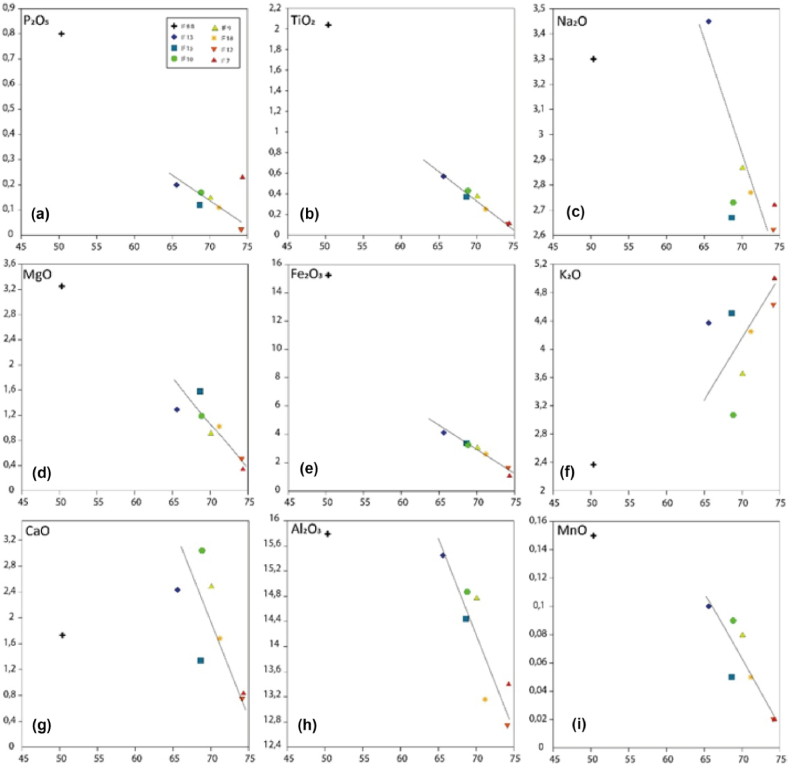
Diagrams of variation of major elements (wt.%) as a function of SiO_2_ (wt.%).

The binary diagrams of the major elements (Fig. 8a–i) as a function of silica show a negative correlation between P_2_O_5_ (Fig. 8a) and TiO_2_ (Fig. 8b), Na_2_O (Fig. 8c), MgO (Fig. 8d), Fe_2_O_3_ (Fig. 8e), CaO (Fig. 8g), Al_2_O_3_ (Fig. 8h), and MnO (Fig. 8i). On the other hand, K_2_O (Fig. 8f) is positively correlated with SiO_2_, expressing the enrichment of the facies in the latter, following petrographic observations.

The geochemical data indicate a negative correlation linking silica to loss on ignition. This is probably related to the leaching of silica during secondary processes. The highest value is for sample IF7 corresponding to porphyry granite rich in quartz minerals. While the lowest value is for sample IF8b and corresponds to quartzite. The value above 70 % is associated with quartz-rich granites. These are acidic magmatic rocks with a very high average silica content. Some rocks, the richest in quartz minerals, show a silica content higher than 74 % (IF12). The Fe_2_O_3_ contents are very contrasted, they varied respectively from 15.24 to 1.07 wt%. The Fe_2_O_3_ content for these samples is inversely proportional to the silica content (Fig. 8e). The highest value is for the monzogranite and corresponds to 15.24 wt% while the other rocks show values lower than 4 wt%. The Al_2_O_3_ contents are relatively low, particularly for the basic rocks where the content reaches 15 wt%. This high alumina content reflects the abundance of plagioclase and chlorite phenocrysts in these rocks, which are generally porphyry in texture. In the microgranite, this content can reach 12.74 wt%. CaO contents are weakly represented, with values varying between 3.04 and 0.74 wt%. The porphyroid granodiorite shows the highest content (3.04 wt%), while the microgranite represents the lowest value. These contents do not show any correlation with the development of the silica content. Observation of the chemical element contents shows a low MgO content in the facies studied, with proportions varying between 0.34 wt% for the porphyry granite and 3.25 wt% for the monzogranite. An increase in alkaline content is noted for the intermediate and acidic terms. For the basic monzodiorite, the lowest K_2_O value is noted, compensated by a contribution of Na_2_O. This is translated in thin section by an albitization of the plagioclases. As for the Fe_2_O_3_ content. We note a decrease in TiO_2_ from the basic and intermediate terms to the most acidic terms (negative correlation). All values are very low, below 0.57 wt%, except for sample IF 8B which has a content of 2.04 wt%. There is a decrease in Na_2_O from the basic and intermediate terms to the more acidic terms (negative correlation). All the values vary little, from 3.3 for the monzodiorite to 2.62 wt% for the monzogranite. The contents of MnO, P_2_O_5,_ and SO_3_ are negligible; they do not exceed 1 wt%.

#### 4.3.2 Detrital and volcano-detrital rocks

**Fig. 9:**
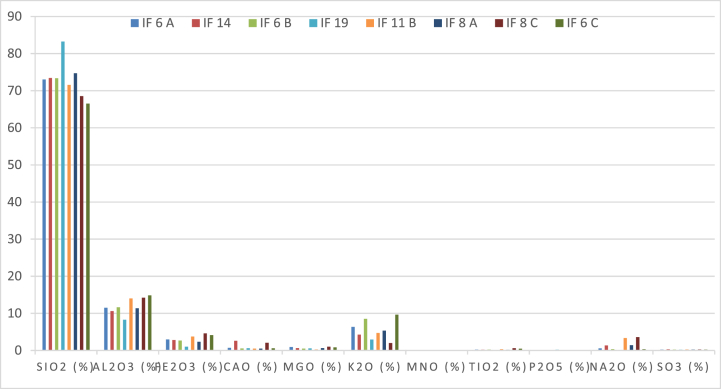
Chemical composition in major elements of detrital and volcano-detrital rocks.

The contents of MnO, P_2_O_5,_ SO_3,_ and TiO_2_ are low; they do not exceed 0.6 wt% (Fig. 9). SiO_2_ contents are relatively high compared to magmatic rocks; they vary between 66.52 % and 83.24 wt%. This silica richness reflects the abundance of quartz phenocrysts. Silica contents show no correlation with loss on ignition (LOI). In particular, the detrital rocks show high contents compared to the volcano-detrital rocks, except for sample IF8a which shows a silica content of 74.7 wt%. Al_2_O_3_ contents are low, especially for the volcano-detrital rocks which show values above 14 wt% except for sample IF8a with a content of 11.36 wt%. The Al_2_O_3_ content shows a negative correlation with SiO_2_. This alumina richness reflects the abundance of plagioclase and chlorite phenocrysts in these general porphyry textured rocks. From the low acidic terms to the most acidic terms, there is an increase in Fe_2_O_3_ content with silica. The values are medium to low, ranging from 4.15 wt% for IF6A to 1.05 wt% for IF19. K_2_O contents are very contrasted. The values varied from 2.02 to 9.64 wt%. The highest contents correspond to samples IF6c, IF6b, and IF6a. The K_2_O content does not show any correlation with either LOI or silica. The Na_2_O contents show little to medium contrast. The values varied between 3.59 and 0.03 wt%. The highest contents correspond to the two samples IF8c and IF11b, their values are above 3 wt%. Samples IF14 and IF8A show values above 1 wt%. The IF19 sample shows a negligible value (less than 0.03 wt%). CaO contents are moderate to weakly represented, with values varying between 0.49 and 2.62 wt%. All samples do not show a correlation with FP or with silica. Sample IF14 shows the highest value, while sample IF 8a shows the lowest CaO.

#### 4.3.3 Carbonate rocks

**Fig. 10:**
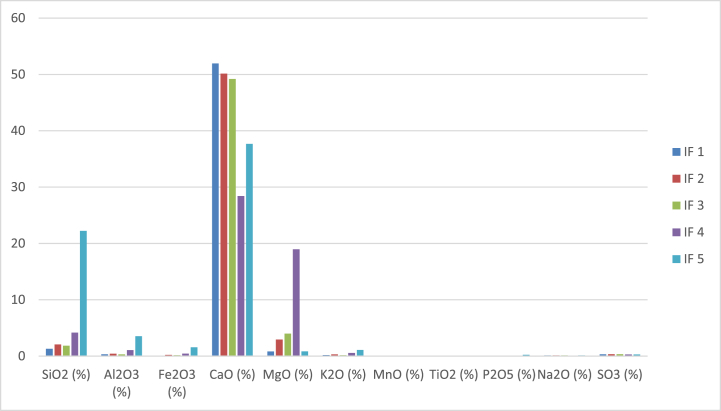
Chemical composition in major elements (in %wt) of different carbonate rocks.

The contents of K_2_O, Na_2_O, MnO, TiO_2_, P_2_O_5,_ and SO_3_ are very low; they do not exceed 1 % (Fig. 10). The SiO_2_ contents are very low and contrasted. The values varied from 1.29 to 22.23 wt%. The highest content corresponds to sample IF5, while the sample with the lowest content corresponds to sample IF1. The SiO_2_ content does not show any correlation with the LOI. All rocks show low Al_2_O_3_ contents, except for sample IF 5 with a content higher than 3.54 wt%. The Al_2_O_3_ contents varied from 0.34 to 3.54 wt%. They show a positive correlation with SiO_2_. The carbonate rocks show an increase in Fe_2_O_3_ content with silica. The values are low, ranging from 0.11 wt% for sample IF1 to 1.56 wt% for sample IF5. The different samples show the presence of a high CaO content. The values varied between 8 and 51.93 wt%. Rocks IF1, IF2, and IF3 show high oxide contents, above 49 %. Rocks IF4 and IF5 show contents below 38 wt%. The SiO_2_ content of the carbonate rocks is very variable. The values varied from 0.82 to 18.97 wt%. The highest content corresponds to sample IF4, while the sample with the lowest content corresponds to sample IF1. The MgO content does not show any correlation with either SiO_2_ or LOI.

## 5 Discussion

### 5.1 Detrital rocks

The geochemical signatures of the RDVDs indicate that they have undergone a moderate to a low degree of chemical weathering. The CIA study also suggests that granitic, granodioritic rocks represent the source provenance which, during weathering and transport, provided the detritus to the supra-crustal units. The CIA is defined as CIA = 100∗ (Al_2_O_3_)/(Al_2_O_3_ + CaO + Na_2_O + K_2_O); where the amount of CaO is that portion which is incorporated in the silicate fraction of the rock and the results of CIA are presented in Table 2. Major trace element data suggest that these rocks are largely derived from felsic igneous rocks, namely granitoids, with a minor contribution from intermediate sources.

**Table 2:** Chemical Index of Alteration (CIA) of the magmatic rocks from the study area.

Sample	Symbol	CIA
Granodiorite	IF 10	62.71615352
Rhyolite	IF 12	61.48648649
Granodiorite	IF 13	60.11673152
Monzogranite	IF 15	62.89198606
Granite	IF 18	60.20128088
Granite	IF 7	61.04783599
Dolerite	IF 8 B	68.08969383
Granite	IF 9	62.10084034

The detrital facies, characterized by large and abrupt variations in strength and poor sorting of angular elements, show the characteristics of slope scree accumulated at the foot of the vigorous reliefs of the newly created collapse faults that cut the Ifni Buttonhole basement. In the rivers, alluvial terraces are made up of gravel, pebbles, and blocks. The materials are sorted according to their size. Their lithological nature is diverse, dolomitic, rhyolitic, granitic, volcanic breccia and tuff, quartzite, and metamorphic sandstone.

**Fig. 11:**
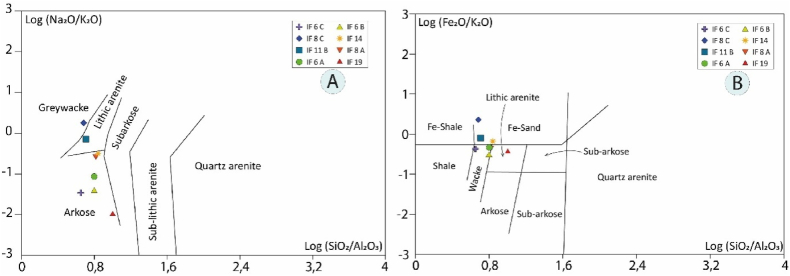
a, Diagram of detrital rock classification of the study area according to Pettijohn et al. [8]; **b**, Diagram of detrital rock classification of the study area [67].

The geochemistry of sedimentary rocks can be used to infer the composition of the source rock, weathering, transport history, and depositional conditions of the sedimentation [62]. Research on the geochemical characteristics of ancient and modern sediments has been carried out by various researchers to infer source rock, provenance, and tectonic setting [3,9,13,48,49,[63], [64], [65], [66]]. The main elemental geochemical parameters have been used to define the tectonic settings of different sedimentary suites [13,63]. Several geochemical classification diagrams have been adopted by several authors such as Pettijohn et al. [8], Folk [6], Herron [67], and Lindsey [68]. In the binary diagram of Pettijohn et al. [8] (Fig. 11a), the samples from the study area belong mainly to the fields arkose (IF 19, IF 16C, IF 6B, IF 6A, IF 8A, IF 14), lithic arenites (IF 18B) and grauwackes (IF 18C). On the log (SiO_2_/Al_2_O_3_)-log (Fe_2_O_3_/K_2_O) classification diagram [67] (Fig. 11b), used for primary rock classification, most samples occupy the fields of sandstone (IF 6A, IF 6C, IF), lithic arenite (IF 8C, IF 11B, IF 14), and Fe-rich sand (IF 19). The high SiO_2_ concentration and medium Al_2_O_3_ contents of the sediments correspond to a low abundance of shale and/or clay components.

**Fig. 12:**
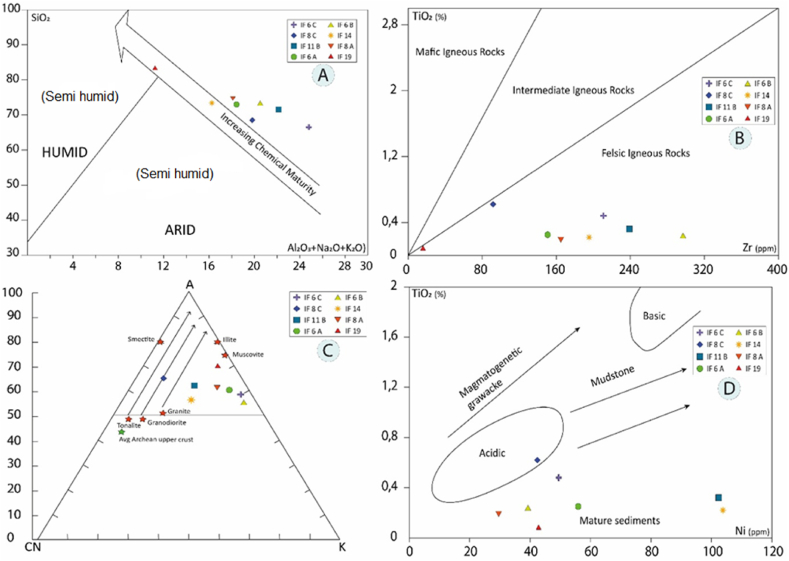
a, Geochemical maturity diagram of different rocks studied [69]; **b**, TiO_2_ variation diagram in the function of Zr [70]; **c**, Ternary diagram A–CN–K (Al_2_O_3_– CaO*+Na_2_O–K_2_O) according to Nesbitt and Young [76]. The arrows show the alteration trend [77]; **d**, TiO_2_ variation diagram in the function of Ni [78].

A bivariate plot of total SiO_2_ vs. total Al_2_O_3_ + K_2_O + Na_2_O proposed by Suttner and Dutta [69] was used to identify the climatic conditions for the detrital rocks. Except for sample IF19, all detrital and volcano-detrital rocks in the study area are formed under semi-arid conditions. Sample IF19 was formed under semi-wet conditions and has the highest degree of maturity of all the samples (Fig. 12a).

Geochemical studies (Table 3) on various samples exposed around the Ifni buttonhole indicate their sub-arkosic to sub-lithic arenite composition which has undergone a moderate degree of alteration. This is also supported by petrographic observations which indicate the low amount of visible feldspar grains and low K, Na, and Ca contents. Moderate alteration is also indicated by high SiO_2_/Al_2_O_3_ ratios indicating a mature nature and probably indicating stable depositional conditions.

**Table 3:** Major and trace elements representative of different compositions of studied rocks.

Samples ------- Major elements (wt.%) and Trace elements (ppm)	IF 4	IF 1	IF 3	IF 2	IF 5	IF 16	IF 8b	IF 15	IF 13	IF 18	IF 7	IF 10	IF 9	IF 12	IF 14	IF 8c	IF 6a	IF 19	IF 11a	IF 8a	IF 6c	IF 6b	IF 11b	IF 17
SiO_2_	4.18	1.29	1.86	2.08	22.23	46.05	50.33	68.64	65.6	71.19	74.33	68.84	70.09	74.16	73.41	68.52	72.99	83.24	71.18	74.7	66.52	73.35	71.57	96.84
Al_2_O_3_	1.06	0.34	0.32	0.44	3.54	15.78	15.79	14.44	15.45	13.16	13.4	14.87	14.78	12.74	10.61	14.21	11.52	8.29	13.28	11.36	14.83	11.65	14.03	0.79
Fe_2_O_3_	0.45	0.11	0.16	0.22	1.56	13.77	15.24	3.38	4.12	2.61	1.07	3.26	3.09	1.6	2.82	4.62	2.96	1.05	3.22	2.34	4.15	2.66	3.76	0.11
CaO	28.41	51.93	49.19	50.15	37.67	3.01	1.73	1.34	2.43	1.68	0.83	3.04	2.49	0.74	2.62	2.06	0.7	0.63	2.36	0.49	0.6	0.54	0.51	<0.10
MgO	18.97	0.82	4	2.95	0.84	9.27	3.25	1.58	1.29	1.02	0.34	1.19	0.92	0.5	0.65	1.03	0.92	0.58	1.02	0.65	0.84	0.5	0.27	0.1
K_2_O	0.57	0.2	0.17	0.31	1.09	1.16	2.37	4.51	4.37	4.25	5	3.07	3.66	4.62	4.28	2.02	6.36	2.93	2.85	5.36	9.64	8.53	4.71	<0.01
MnO	0.05	0.01	0.01	0.01	0.1	0.22	0.15	0.05	0.1	0.05	0.02	0.09	0.08	0.02	0.02	0.07	0.01	0.03	0.06	0.02	0.03	0.01	0.01	<0.01
TiO_2_	0.03	0.01	0.01	0.01	0.12	2.65	2.04	0.37	0.57	0.25	0.11	0.43	0.38	0.1	0.22	0.62	0.25	0.08	0.39	0.19	0.48	0.24	0.32	0.02
P_2_O_5_	0.04	0.02	0.02	0.02	0.23	0.38	0.8	0.12	0.2	0.11	0.23	0.17	0.15	0.02	0.07	0.14	0.07	0.23	0.1	0.05	0.11	0.03	0.07	0.02
Na_2_O	0.08	0.13	0.13	0.13	0.13	2.6	3.3	2.67	3.45	2.77	2.72	2.73	2.87	2.62	1.36	3.59	0.55	0.03	2.68	1.39	0.33	0.34	3.36	0.03
SO_3_	0.3	0.33	0.35	0.35	0.28	0.23	0.25	0.3	0.25	0.23	0.35	0.4	0.25	0.28	0.3	0.28	0.25	0.22	0.35	0.25	0.25	0.25	0.28	0.08
As	56	27	53	21	57	89	45	83	30	64	106	46	40	69	64	64	53	67	66	74	46	68	85	<8
B	45	37	39	33	46	63	68	44	39	30	34	32	37	29	37	43	64	33	32	33	49	43	41	0
Ba	132.1	8.6	24.5	31.7	305.4	323.3	501.8	793.8	846.8	581.4	251.1	584.7	731.6	788.1	1157.6	458.2	486.3	303.7	724.5	616.1	923	1185.8	1091.7	115
Be	0.1	<0.1	<0.1	<0.1	0.3	0.3	1.8	1.8	1.9	2.7	1.9	2	1.1	2.1	2.5	0.9	1.7	1.2	0.9	1.6	1.4	0.3	1.3	0.1
Bi	<0.1	<0.1	<0.1	<0.1	<0.1	<0.1	<0.1	<0.1	<0.1	<0.1	<0.1	<0.1	<0.1	<0.1	<0.1	<0.1	<0.1	<0.1	<0.1	<0.1	<0.1	<0.1	<0.1	<0.1
Cd	<0.1	<0.1	<0.1	<0.1	<0.1	<0.1	<0.1	<0.1	<0.1	<0.1	<0.1	<0.1	<0.1	<0.1	<0.1	<0.1	<0.1	<0.1	<0.1	<0.1	<0.1	<0.1	<0.1	<0.1
Co	85.6	48.2	71.7	112.6	342.8	114	49.4	110.4	28.1	16.5	120.3	384.8	126.1	87.8	35	46.8	44.6	22.1	56.6	71.6	76.3	60.3	39	27
Cr	17.7	20.3	17	14.1	28.5	228.6	25	77.4	51.7	92.3	49.1	65.3	69.4	121.8	49.1	62.4	393.1	61.4	67.6	53.9	69.1	58	112.9	264.6
Cu	4.5	0.2	2.4	2.8	0.1	<0.1	<0.1	23.4	8.5	9.6	5.6	5	6.5	6.3	8.9	3	6.6	4.9	14.2	13.8	6.2	14.4	5.3	7
Ge	<10	<10	<10	<10	<10	<10	<10	<10	<10	<10	<10	<10	<10	<10	<10	<10	<10	<10	<10	<10	<10	<10	<10	<10
Li	4.2	<0.1	<0.1	<0.1	8.3	97.7	55.8	31.2	15.9	11.6	6.7	9	5.3	1.2	15.7	8.5	21.3	2	4.1	18.9	29.7	6.8	5.9	<0.1
Mo	0.7	1.4	1.3	1	1.9	2	3.2	1.9	1.5	1.9	1.4	0.4	2.6	2.3	<0.1	1.8	2	2	0.9	1.7	2	1.6	1.7	2.7
Nb	<0.1	<0.1	<0.1	<0.1	<0.1	12	31.1	0.4	8.5	<0.1	0.7	6.4	5.6	7.8	6.5	3.7	10	<0.1	1.3	13.4	7.8	3	10	<0.1
Ni	32.9	23.7	31.9	39	36.2	171	37.2	44.4	49.5	34.5	25.8	36.1	38.7	38	103.8	42.4	55.9	42.8	162.8	29.6	49.5	39.3	102.4	48.8
Pb	10.4	5.1	31.1	15.1	8.2	14.6	3.5	15	29.3	7.9	14.7	9.2	10.7	3.7	16.4	21.1	4.7	5.8	23.5	10.1	4.1	7.3	12.7	10.8
Sb	<0.1	7.2	7.3	12.8	3.2	18.7	5.4	3.9	8.4	5.2	5	11.9	9.6	6.7	7.8	<0.1	1.9	14.4	6.5	5.7	4.5	5.9	6.8	11.7
Se	<0.1	<0.1	<0.1	<0.1	<0.1	<0.1	<0.1	<0.1	<0.1	<0.1	<0.1	<0.1	<0.1	<0.1	<0.1	<0.1	<0.1	<0.1	<0.1	<0.1	<0.1	<0.1	<0.1	<0.1
Sn	<0.1	<0.1	<0.1	<0.1	<0.1	<0.1	<0.1	2	<0.1	<0.1	<0.1	<0.1	<0.1	<0.1	<0.1	<0.1	<0.1	<0.1	<0.1	<0.1	<0.1	<0.1	<0.1	<0.1
Sr	60.8	144.5	97.3	233.6	77.9	141	66.5	145.5	227.6	141.5	70.1	297.5	203.7	86.4	48.9	205.3	14	24.4	222.9	24	39.8	60.3	97.5	9.5
V	11	<0.1	3.1	1.1	16.1	298.3	106.8	24.8	30.2	17.2	0.9	28.7	25	0.5	6.1	32.5	7.4	3.3	27.9	1.4	5.8	2.9	1.1	2.6
W	<23	<23	<23	<23	<23	<23	<23	<23	<23	<23	<23	<23	<23	<23	<23	<23	<23	<23	<23	<23	<23	<23	<23	<23
Y	2.8	2	1.9	1.5	14.4	26.4	35.1	11.4	22.8	16.7	6.2	14.1	12.2	10.2	33.3	16.4	16.2	3.5	13.9	21.9	22.7	15.1	25.9	0.8
Zn	86.1	53.4	63.5	110.2	50.6	324.9	115.7	72.1	108	49.1	28.9	85.5	55.1	32.4	48.2	61	39.5	15.1	83.2	44.5	29.5	27	23.6	21.4
Zr	8.7	6	30.2	6.1	21	89.7	113.6	52	58.9	25.4	87.9	70.3	52.2	62.2	195.4	91.7	150.6	15.9	51.6	164.9	210.9	296.9	239	18.1
PF	44.68	43.6	43.6	42.66	30.89	4.6	3.05	2.27	1.29	1.24	0.69	0.64	0.59	0.32	2.68	2	1.34	1.22	0.95	0.91	0.79	0.59	0.28	0.14

Zr concentrations are also used to characterize the nature and composition of source area rocks [70]. The average Zr and Ti concentrations do not show a significantly wide variation. The samples do not show a correlation between Zr and TiO_2_, suggesting the presence in varying proportions of some accessory minerals such as zircon, rutile, and ilmenite. A TiO_2_ vs Zr plot distinguishes three different types of rocks from the source zone, i.e. felsic. The TiO_2_ vs Zr graph of the Hayashi et al. [70] shales (Fig. 12b) represents mainly felsic igneous rocks and partially intermediate source zone rocks.

In the Al_2_O_3_–CaO*+Na_2_O–K_2_O (A–CN–K) diagram, the majority of samples (IF14, IF18B, IF8A, IF16A, IF6C, IF6B, IF19) cluster along the Al_2_O_3_–K_2_O (A-K) line between the kaolinite-muscovite-biotite field, which also indicates the tendency towards granite alteration, represented at the base of the plagioclase-K-feldspar line. Two samples (F14 and IF18B) fall between the A-CN and A-K lines closer to the muscovite and potassium feldspar field. These two samples are parallel to the granite alteration line. Only one sample is in the granodiorite alteration line (Fig. 12c). The A–CN–K diagram (Fig. 12c) indicates granitic and granodioritic source rocks that may have been supplied by exposures in the study area. A felsic and recycled source for these QPC-quartzites is also inferred by the ratios of Zr to TiO_2_ which also indicate moderate alteration and reasonable enrichment of zircon during sediment recycling processes.

According to the Ni (ppm) vs TiO_2_ binary diagram (Fig. 12d), all the analyzed samples belong mainly to the felsic zone field.

### 5.2 Magmatic rocks

**Fig. 13:**
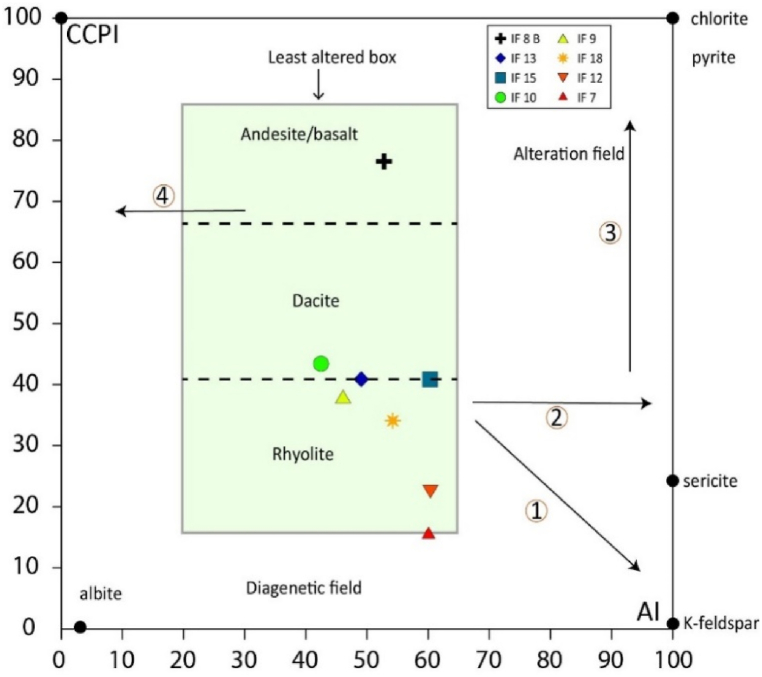
Alteration diagram according to Large et al. [71].

Alteration indices can be ratios of selected mobile major elements representative of an alteration mineral of interest or a primary mineral destabilized by the alteration process. These indices are easy to calculate and are popular with exploration geologists. From the examination of the major oxide compositions of all the magmatic samples analyzed, a variation in LOI values can be seen ranging from 0.32 to 3.05. The highest value is for sample IF 8B rich in 3.05 while the lowest value corresponds to sample IF 7. In general, the losses of fire for magmatic rocks are lower compared to detrital and carbonate rocks. Element mobility was assessed from the alteration diagram of Large et al. [71], which combines the Ishikawa alteration index [AI = 100 × (K_2_O + MgO)/(K_2_O + MgO + Na_2_O + CaO)] and the chlorite-carbonate-pyrite index [CCPI = 100 × (FeO + MgO)/(FeO + MgO + Na_2_O + CaO)]. All samples, except for IF 7, plot on the least altered box (Fig. 13) which suggests that their major element compositions are reliable for geochemical examination [71].

**Fig. 14:**
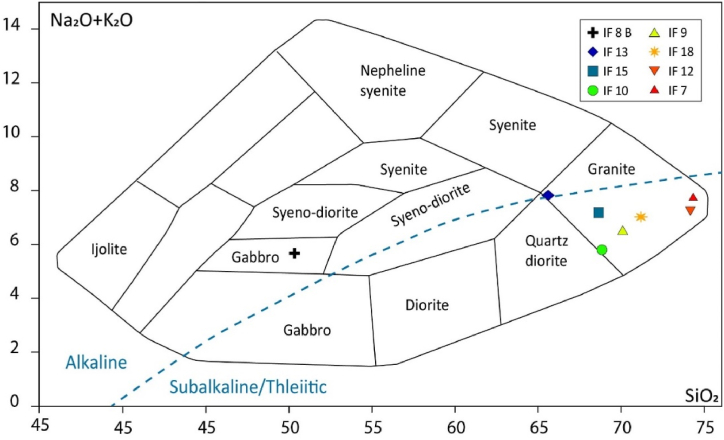
Classification diagram of igneous rocks, according to Cox et al. [72].

Many diagrams are presented in the literature for the characterization of magmatic rocks. To determine the nature of magmatic rocks, we have chosen the diagram of Cox et al. [72], which seems to us the most appropriate for the rocks studied. We also noted that despite the perturbations induced on certain chemical elements by the alteration processes, their effects remain globally limited on the rock compositions, which allows us to approach this with a large margin of confidence. The magmatic rocks of the Ifni buttonhole have mafic and felsic compositions, with silica contents ranging from 65.6 % to 74.33 % for the granitoids, and 50.33 % for the dolerite dyke. In addition to classification, the Cox et al. [72] diagram also has the property of discriminating between alkaline and sub-alkaline series. The magmatic rocks of the Ifni buttonhole show not only the subalkaline trend for the granitoids but also an alkaline trend for the dolerite dyke (Fig. 14).

### 5.3 Carbonate rocks

**Fig. 15:**
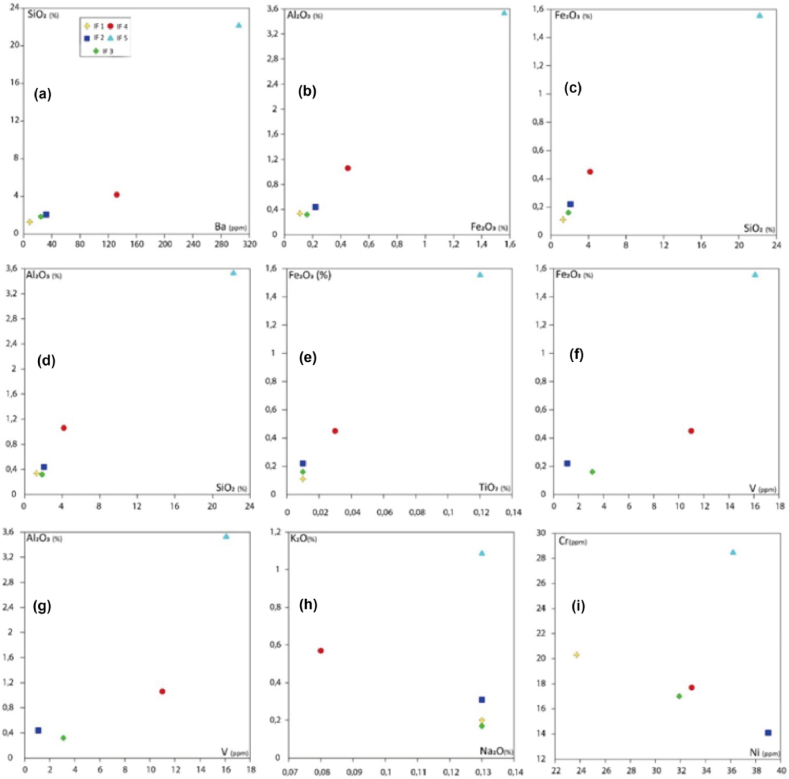
Binary diagrams of major (wt.%) and trace (ppm) elements in different carbonate rocks.

The lack of correlation between Na_2_O and K_2_O and the variation of their values depend almost exclusively on the presence of feldspars and, to a lesser extent, phyllosilicate minerals (muscovite), which are mainly identified in different samples (Fig. 15). In general, it is observed that SiO_2_ increases with the enrichment of Al_2_O_3_ and Fe_2_O_3_t, as shown in Fig. 15a, b, c. A positive trend is noted for the Al_2_O_3_ and Fe_2_O_3_t contents (Fig. 15b). These three major element oxides are included in the different mineral phases (epidote, quartz, and chlorite) found in the IF4 and IF5 samples. However, it should also be considered that the above-mentioned element contents may also depend on the presence of other mineral phases, even if they are present in rather low quantities. In particular, SiO_2_ and Al_2_O_3_ contents may be affected by the inclusion of feldspars and quartz (Fig. 15d), while Fe_2_O_3_t tends to be higher in samples containing Fe oxides. The presence of accessory ilmenite in the carbonate samples probably contributes to their Fe_2_O_3_t, TiO_2,_ and V contents (Fig. 15e and f); however, the positive correlation between Al_2_O_3_ and V (Fig. 15g) suggests that the V content may also be associated with the modal composition of the epidote. For Mn, it seems that some samples have slightly higher values, probably attributed to the layers of mica crystals between the calcitic crystals (Fig. 15h and i).

**Fig. 16:**
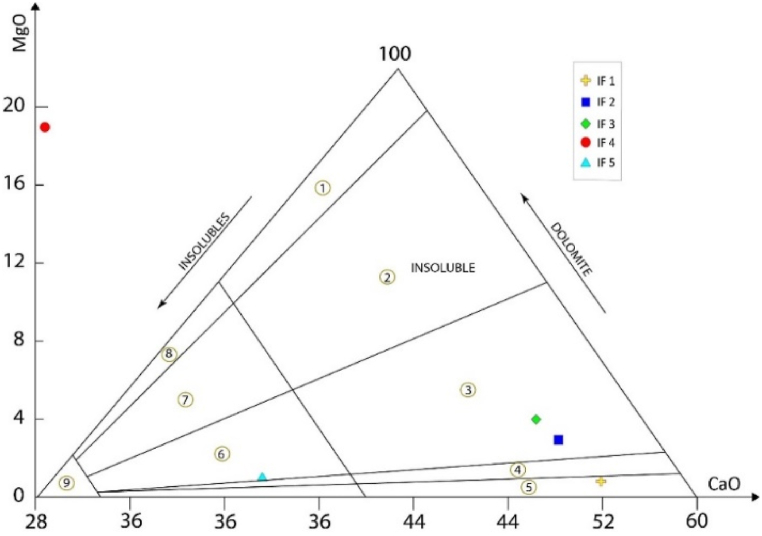
Geochemical nomenclature of carbonate rocks, according to Martinet and Sougy [73]: 1, dolostone; 2, calcareous dolostone; 3, dolomitic limestones; 4, magnesian limestone; 5, limestone; 6, calcaro-dolomitic chert; 7, dolomito-calcareous chert; 8, dolomitic chert; 9, chert.

The major elements (Ca, Mg) were measured on the whole sample. The Ca and Mg contents were converted into CaO (%) and MgO (%) to characterize the chemical natures of the carbonate samples, following the classification of Martinet and Sougy [73]. Using this diagram, it can be seen that the Lakhssas Plateau samples do not represent a wide variety of facies: dolomitic limestone, limestone, and calcaro-dolomitic chert (Fig. 16). These facies outcrop in distinct metric horizons within the different carbonate sequences studied.

**Fig. 17:**
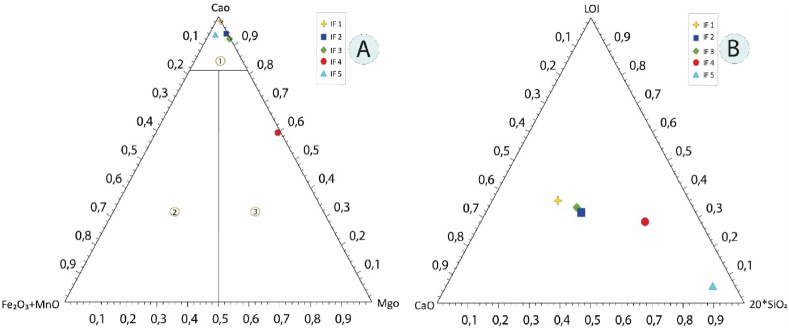
a, Ternary classification diagram of carbonate rocks: 1. calciocarbonatite, 2. ferrocarbonatite, 3. Magnesiocarbonatite; **b**, Ternary diagram CaO-20*SiO_2_-LOI, showing the relationship between SiO_2_ content and CaO/LOI ratio [74].

The total rock geochemical composition and in particular their CaO/MgO ratio allow these rocks to be distinguished into two main types, irrespective of their metamorphic and/or metasomatic grade [74]. All rocks are classified as calciocarbonatites, while sample IF4 falls into the magnesiocarbonatite domain. Based on the ternary graph in Fig. 17a and b, the calcitic and dolomitic samples show a linear increase in SiO_2_, regardless of their CaO/LOI ratio values, which remain relatively constant. The highest SiO_2_ contents are observed in the calc-dolomitic chert.

## 6 Conclusion and outlook

Through this paper, we have carried out the different facies of the Precambrian buttonhole of Sidi Ifni and the Lakhssas Plateau (Western Anti-Atlas). The plutonic rocks described are of variable color, compact, homogeneous, and very hard, difficult to alter. They show not only the sub-alkaline trend for the granitoids but also an alkaline trend for the dolerite dyke. The geochemical data indicate a negative correlation linking silica to loss on ignition. This is probably related to the leaching of silica during secondary processes. The majority of the samples behave as less altered, suggesting that their major element compositions are reliable for geochemical examinations. These granitoids are excellent deposits for ornamental and building rocks. The following points summarize the results of this contribution.

• The geochemical attributes of the RDVDs imply a degree of chemical weathering ranging from moderate to low. Additionally, the CIA analysis suggests that granitic and granodioritic rocks probably acted as the primary source materials, supplying detritus to the supra-crustal units through weathering and transport mechanisms.
• The calcitic and dolomitic samples show a linear increase in SiO_2_, regardless of their CaO/LOI ratio values, which remain relatively constant.
• The highest SiO_2_ contents are observed in the calc-dolomitic chert. These facies outcrop in distinct metric horizons within the different carbonate sequences studied.
• Limestones and dolomites represent materials that can be used in the building sector for all types of concrete.
• Lower Cambrian dolomites and limestones can be exploited for aggregates. The types of carbonate rock exploited are pink dolomites and all Cambrian limestones. The samples are valued according to their attractive surface and color. High SiO_2_ contents are observed in all samples.
• Geochemical studies of RDVD from the Ifni buttonhole were carried out to determine their provenance, compositional maturity, and degree of alteration. All major oxides decreased as silica content increased, and a negative linear relationship of SiO_2_ with Al_2_O_3_ indicated that the major element composition of these rocks was largely controlled by the amount of quartz. Most of the samples are formed under semi-arid conditions and show the greatest degree of maturity under stable depositional conditions. A felsic and recycled source is inferred for these RDVDs, also indicating moderate alteration and reasonable zircon enrichment during sediment recycling processes.

## Data availability statement

The current manuscript has no data associated that has been deposited into a publicly available repository. Data may be made available on reasonable request.

## Funding

Open access funding is provided by University of Galway.

## CRediT authorship contribution statement

**Mohamed Mahmoud Sebbab:** Writing – original draft, Software, Resources, Methodology, Investigation, Formal analysis, Data curation, Conceptualization. **Mehdi Ousbih:** Writing – original draft, Validation, Software, Resources, Methodology, Investigation, Formal analysis, Data curation, Conceptualization. **Mohamed En-Nasiry:** Writing – original draft, Validation, Formal analysis. **Abdelhadi El Ouahidi:** Writing – original draft, Visualization, Validation, Supervision. **Kamal Abdelrahman:** Writing – review & editing, Validation, Funding acquisition, Formal analysis. **Abdessamad El Atillah:** Writing – original draft, Visualization, Formal analysis. **Md Galal Uddin:** Writing – review & editing, Validation, Funding acquisition, Formal analysis. **Armel Zacharie Ekoa Bessa:** Writing – original draft, Visualization, Validation, Methodology, Formal analysis. **Mohammed S. Fnais:** Writing – review & editing, Visualization. **Agnieszka I. Olbert:** Writing – review & editing, Visualization, Validation. **Mohamed Abioui:** Writing – review & editing, Visualization, Validation.

## Declaration of competing interest

The authors declare that they have no known competing financial interests or personal relationships that could have appeared to influence the work reported in this paper.

## References

[1] Ive G.J., Gruneberg S.L. (2000).

[2] Dickinson W.R., Suczek C.A. (1979). Plate tectonics and sandstone compositions. AAPG Bull..

[3] Roser B.P., Korsch R.J. (1988). Provenance signatures of sandstone-mudstone suites determined using discriminant function analysis of major-element data. Chem. Geol..

[4] Ambassa Bela V., Ekoa Bessa A.Z., Ngueutchoua G., Aonsi Kamani F., Abioui M., Kwewouo Janpou A., Ngueudong Zebaze M.L., Duviol Tsanga A., Armstrong-Altrin J.S. (2022). Petrography and geochemistry of beach sediments along the central coast of Cameroon: constraints on paleoweathering, provenance and tectonic setting. Arabian J. Geosci..

[5] Tsanga A.D., Ekoa Bessa A.Z., Ngueutchoua G., Ngokam G.S., Jacques-David S.M., Ambassa Bela V., Kwewouo Janpou A., Abioui M., Armstrong-Altrin J.S. (2023). Evaluating the characteristics, provenance and weathering conditions of beach sediments from mineralogical and geochemical patterns in the lower part of the Cameroonian coast. J. Afr. Earth Sci..

[6] Folk R.L. (1974).

[7] Pettijohn F.J. (1975).

[8] Pettijohn F.J., Potter P.E., Siever R. (1972).

[9] Bhatia M.R., Crook K.A.W. (1986). Trace element characteristics of graywackes and tectonic setting discrimination of sedimentary basins. Contrib. Mineral. Petrol..

[10] Dickinson W.R., Zuffa G.G. (1985). Provenance of Arenites.

[11] Valloni R., Mezzadri G. (1984). Compositional suites of terrigenous deep-sea sands of the present continental margins. Sedimentology.

[12] Floyd P., Leveridge B. (1987). Tectonic environment of the Devonian Gramscatho basin, south Cornwall: framework mode and geochemical evidence from turbiditic sandstones. J. Geol. Soc..

[13] Roser B.P., Korsch R.J. (1986). Determination of tectonic setting of sandstone-mudstone suites using SiO2 content and K2O/Na2O ratio. J. Geol..

[14] Tao H., Sun S., Wang Q., Yang X., Jiang L. (2014). Petrography and geochemistry of Lower Carboniferous greywacke and mudstones in Northeast Junggar, China: implications for provenance, source weathering, and tectonic setting. J. Asian Earth Sci..

[15] Verma S.P., Armstrong-Altrin J.S. (2016). Geochemical discrimination of siliciclastic sediments from active and passive margin settings. Sediment. Geol..

[16] Verma S.P., Armstrong-Altrin J.S. (2013). New multi-dimensional diagrams for tectonic discrimination of siliciclastic sediments and their application to Precambrian basins. Chem. Geol..

[17] Xie Q.F., Cai Y.F., Dong Y.P., Zhai M.G., Li D.P. (2019). Geochemical characteristics of the Permian marine mudstone and constraints on its provenance and paleoenvironment in the Fenghai area, Fujian Province, southeastern China. Petrol. Sci..

[18] Mortaji A., Gasquet D., Ikenne M., Beraaouz E.H., Barbey P., Lahmam M., El Aouli E.H. (2007). The tardi-Pan-African granitoids of South-Westerner Anti-Atlas (Morocco): evolution from magnesian to ferroan type. Example of the Ifni inlier. Estud. Geol..

[19] Ikenne M., Söderlund U., Ernst R.E., Pin C., Youbi N., El Aouli E.H., Hafid A. (2017). A c. 1710 Ma mafic sill emplaced into a quartzite and calcareous series from Ighrem, Anti-Atlas – Morocco: evidence that the Taghdout passive margin sedimentary group is nearly 1 Ga older than previously thought. J. Afr. Earth Sci..

[20] Sebbab M.M., El Ouahidi A., Ousbih M., Ouboulahcen S., Abdelrahman K., Abioui M. (2023). Integrated geotechnical approach and GIS for identification of geological resources exploitable quarries for sustainable development in Ifni inlier and Lakhssas Plateau (western Anti Atlas, Morocco). Appl. Sci..

[21] Ikirri M., Jaffal M., Rezouki I., Echogdali F.Z., Boutaleb S., Abdelrahman K., Abu-Alam T., Faik F., Kchikach A., Abioui M. (2023). Contribution of gravity data for structural characterization of the Ifni inlier, western Anti-Atlas, Morocco: hydrogeological implications. Appl. Sci..

[22] Benziane F., Yazidi A., Schulte B., Boger S., Stockhammer S., Lehmann A., Saadane A., Yazidi M. (2016). Carte géologique du Maroc, feuille au 1/50 000 Tlata Al Akhçaç. Notes Mém. Serv. Géol. Maroc.

[23] Critelli S. (2018). Provenance of Mesozoic to Cenozoic Circum-Mediterranean sandstones in relation to tectonic setting. Earth Sci. Rev..

[24] Critelli S., Martín-Martín M. (2022). Provenance, Paleogeographic and paleotectonic interpretations of Oligocene-Lower Miocene sandstones of the western-central Mediterranean region: a review. J. Asian Earth Sci. X.

[25] Critelli S., Martín-Martín M. (2024). History of western Tethys Ocean and the birth of the circum-Mediterranean orogeny as reflected by source-to-sink relations. Int. Geol. Rev..

[26] Boudda A., Choubert G. (1972). Sur la limite inférieure du Cambrien au Maroc. C. R. Acad. Sci..

[27] Rosanov A.Y., Debrenne F. (1974). Age of archeocyathid assemblage. Am. J. Sci..

[28] Schmitt M. (1978). Stromatolites from the Tiout section. Precambrian-Cambrian boundary beds, Anti-Atlas Morocco. Geol. Mag..

[29] Sdzuy K. (1978). The precambrian-cambrian boundary beds in Morocco (preliminary report). Geol. Mag..

[30] Choubert G. (1947). L'accident majeur de l'Anti-Atlas. C. R. Acad. Sci..

[31] Choubert G., Faure-Muret A. (1970). Les corrélations du Précambrien, Anti-Atlas occidental et central. Mém. Serv. Géol. Maroc.

[32] Leblanc M. (1975).

[33] Leblanc M., Lancelot J.R. (1980). Interprétation géodynamique du domaine panafricain (Précambrien terminal) dans l'Anti-Atlas (Maroc) à partir de données géologiques et géochronologiques. Can. J. Earth Sci..

[34] Critelli S., Criniti S., Ingersoll R.V., Cavazza W., Di Capua A., De Rosa R., Kereszturi G., Le Pera E., Rosi M., Watt S.F.L. (2023).

[35] Costamagna L., Criniti S. (2024). Interpreting siliciclastic sedimentation in the upper paleozoic mulargia-escalaplano basin (Sardinia, Italy): influence of tectonics on provenance. J. Palaeogeogr..

[36] Hefferan K., Soulaimani A., Samson S.D., Admou H., Inglis J., Saquaque A., Chaib L., Heywood N. (2014). A reconsideration of Pan African orogenic cycle in the Anti-Atlas Mountains, Morocco. J. Afr. Earth Sci..

[37] Thomas R.J., Fekkak A., Ennih N., Errami E., Loughlin S.C., Gresse P.G., Chevallier L.P., Liégeois J.P. (2004). A new lithostratigraphic framework for the Anti-Atlas Orogen, Morocco. J. Afr. Earth Sci..

[38] Choubert G. (1963). Histoire géologique du Précambrien de l'Anti-Atlas. Notes Mém. Serv. Géol. Maroc162.

[39] Leblanc M. (1981). Ophiolites précambriennes et gîtes arséniés de cobalt (Bou Azzer-Maroc). Notes Mém. Serv. Géol. Maroc.

[40] Villeneuve M., Cornée J.J. (1994). Structure, evolution and paleogeography of the West African craton and bordering belts during the Neoproterozoic. Precambrian Res..

[41] Ennih N., Liégeois J.P. (2001). The Moroccan Anti-Atlas: the West African craton passive margin with limited Pan-African activity. Implications for the northern limit of the craton. Precambrian Res..

[42] Thomas R.J., Chevallier L.P., Gresse P.G., Harmer R.E., Eglington B.M., Armstrong R.A., de Beer C.H., Martini J.E.J., de Kock G.S., Macey P.H., Ingram B.A. (2002). Precambrian evolution of the sirwa window, anti-atlas orogen, Morocco. Precambrian Res..

[43] Gasquet D., Levresse G., Cheilletz A., Azizi-Samir M.R., Mouttaqi A. (2005). Contribution to a geodynamic reconstruction of the Anti-Atlas (Morocco) during Pan-African times with the emphasis on inversion tectonics and metallogenic activity at the Precambrian–Cambrian transition. Precambrian Res..

[44] Gasquet D., Ennih N., Liégeois J.P., Soulaimani A., Michard A., Michard A., Saddiqi O., Chalouan A., Frizon de Lamotte D. (2008). Continental Evolution: the Geology of Morocco.

[45] Blein O., Baudin T., Soulaimani A., Cocherie A., Chévremont P., Admou H., Ouanaimi H., Hafid A., Razin P., Bouabdelli M., Roger J. (2014). New geochemical, geochronological and structural constraints on the ediacaran evolution of the south sirwa, agadir melloul and iguerda inliers, anti-atlas, Morocco. J. Afr. Earth Sci..

[46] Baidada B., Cousens B., Alansari A., Soulaimani A., Barbey P., Ilmen S., Ikenne M. (2017). Geochemistry and Sm-Nd isotopic composition of the Imiter Pan-African granitoids (Saghro massif, eastern Anti-Atlas, Morocco): geotectonic implications. J. Afr. Earth Sci..

[47] Benziane F., Yazidi A. (1982). Géologie de la boutonnière d'Ifni (Anti-Atlas occidental, Maroc). Notes Mém. Serv. Géol. Maroc.

[48] Perri F., Critelli S., Martín-Martín M., Montone S., Amendola U. (2017). Unravelling hinterland and offshore palaeogeography from pre-to-syn-orogenic clastic sequences of the Betic Cordillera (Sierra Espuña), Spain. Palaeogeogr. Palaeoclimatol. Palaeoecol..

[49] Perri F., Martin-Martin M., Maaté A., Hlila R., Maaté S., Criniti S., Capobianco W., Critelli S. (2022). Provenance and paleogeographic implications for the cenozoic sedimentary cover of the ghomaride complex (internal rif belt), Morocco. Mar. Petrol. Geol..

[50] Critelli S., Perri F., Arribas J., Herrero M.J., Ingersoll R.V., Lawton T.F., Graham S. (2018).

[51] Critelli S., Martín-Martín M., Capobianco W., Perri F. (2021). Sedimentary history and palaeogeography of the cenozoic clastic wedges of the malaguide complex, internal betic cordillera, southern Spain. Mar. Petrol. Geol..

[52] Kairouani H., Zaghloul M.N., Abbassi A., Micheletti F., Fornelli A., El Mourabet M., Piccoli F., Criniti S., Critelli S. (2023). Provenance and source-to-sink of lower-middle Jurassic sediments from Hinterland mounts to NW-Gondwana hyper-extended passive margin (Prerif sub-domain, External Rif, Morocco): first evidence from sedimentary petrology and detrital zircon geochronology. Mar. Petrol. Geol..

[53] Kairouani H., Abbassi A., Zaghloul M.N., El Morabet M., Micheletti F., Fornelli A., Mongelli G., Critelli S. (2024). The Jurassic climate change in the northwest Gondwana (External Rif, Morocco): evidence from geochemistry and implication for paleoclimate evolution. Mar. Petrol. Geol..

[54] Benziane F. (1974).

[55] Yazidi A. (1976).

[56] Charlot R. (1982). Caractérisation des événements éburnéens et panafricains dans l'Anti-Atlas marocain; apport de la méthode géochronologique Rb-Sr. Notes Mém. Serv. Géol. Maroc.

[57] Lahmam M., Beraaouz E.H. (1999). Les granitoïdes panafricains de la boutonnière d'Ifni : Marqueurs de la transition d’un contexte de collision à un autre anorogénique. Notes Mem. Serv. Géol. Maroc.

[58] Soulaimani A., Bouabdelli M. (2005). Le plateau de Lakhssas (Anti Atlas Occidental, Maroc): Un graben fini-précambrien réactivé à l’hercynien. Ann. Soc. Geol. Nord.

[59] Segard M. (2013).

[60] Best M. (2003).

[61] Marsaglia K.M., Barone M., Critelli S., Busby C., Fackler-Adams B. (2016). Petrography of volcaniclastic rocks in intra-arc volcano-bounded to fault-bounded basins of the Rosario segment of the Lower Cretaceous Alisitos oceanic arc, Baja California, Mexico. Sediment. Geol..

[62] McLennan S.M. (1993). Weathering and global denudation. J. Geol..

[63] Bhatia M.R. (1983). Plate tectonics and geochemical composition of sandstones. J. Geol..

[64] Nesbitt H.W., Young G.M., McLennan S.M., Keays R.R. (1996). Effects of chemical weathering and sorting on the petro genesis of siliclastic sediments with implications for provenance studies. J. Geol..

[65] Barbera G., Critelli S., Mazzoleni P. (2011). Petrology and geochemistry of Cretaceous sedimentary rocks of the Monte Soro Unit (Sicily, Italy): constraints on weathering, diagenesis and provenance. J. Geol..

[66] Corrado S., Aldega L., Perri F., Critelli S., Muto F., Schito A., Tripodi V. (2019). Detecting syn-orogenic and sediment provenance of the Cilento wedge top basin (southern Apennines, Italy) by mineralogy and geochemistry of fine grained sediments and petrography of dispersed organic matter. Tectonophysics.

[67] Herron M.M. (1988). Geochemical classification of terrigenous sands and shales from core or log data. J. Sediment. Petrol..

[68] Lindsey D.A. (1999).

[69] Suttner L.J., Dutta P.K. (1986). Alluvial sandstone composition and paleoclimate, I. Framework mineralogy. J. Sediment. Petrol..

[70] Hayashi K., Fujisawa H., Holland H., Ohmoto H. (1997). Geochemistry of ∼1.9 Ga sedimentary rocks from northeastern Labrador Canada. Geochem. Cosmochim. Acta.

[71] Large R.R., Gemmell J.B., Paulick H. (2001). The alternation box plot: a simple approach to understanding the relationship between alteration mineralogy and lithogeochemistry associated with volcanic-hosted massive sulfide deposits. Econ. Geol..

[72] Cox K.G., Bell J.D., Pankhurst R.J. (1979).

[73] Martinet B., Sougy J. (1961). Utilisation pratique des classifications chimiques des roches carbonatées. Ann. Fac. Sci. Univ. Dakar.

[74] Woolley A.R., Kempe D.R.C., Bell K. (1989). Carbonatites, Genesis and Evolution.

[75] Benziane F., Yazidi A., Schulte B., Boger S., Stockhammer S., Lehmann A., Saadane A., Yazid M. (2016). Notice explicative de la carte géologique du Maroc au 1/50000, Feuille Sidi Ifni. Notes Mém. Serv. Géol. Maroc.

[76] Nesbitt H.W., Young G.M. (1984). Prediction of some weathering trends of plutonic and volcanic rocks based on thermodynamic and kinetic considerations. Geochem. Cosmochim. Acta.

[77] Condie K.C. (1993). Chemical composition and evolution of the upper continental crust: contrasting results from surface samples and shales. Chem. Geol..

[78] Floyd P.A., Winchester J.A., Park R.G. (1989). Geochemistry and tectonic setting of lewisian clastic metasediments from the early proterozoic loch maree group of gairloch, NW Scotland. Precambrian Res..

